# Characterization of Chromium Compensated GaAs Sensors with the Charge-Integrating JUNGFRAU Readout Chip by Means of a Highly Collimated Pencil Beam

**DOI:** 10.3390/s21041550

**Published:** 2021-02-23

**Authors:** Dominic Greiffenberg, Marie Andrä, Rebecca Barten, Anna Bergamaschi, Martin Brückner, Paolo Busca, Sabina Chiriotti, Ivan Chsherbakov, Roberto Dinapoli, Pablo Fajardo, Erik Fröjdh, Shqipe Hasanaj, Pawel Kozlowski, Carlos Lopez Cuenca, Anastassiya Lozinskaya, Markus Meyer, Davide Mezza, Aldo Mozzanica, Sophie Redford, Marie Ruat, Christian Ruder, Bernd Schmitt, Dhanya Thattil, Gemma Tinti, Oleg Tolbanov, Anton Tyazhev, Seraphin Vetter, Andrei Zarubin, Jiaguo Zhang

**Affiliations:** 1 PSD Detector Group, Paul Scherrer Institut (PSI), Forschungsstrasse 111, CH-5232 Villigen PSI, Switzerland; marie.andrae@psi.ch (M.A.); rebecca.barten@psi.ch (R.B.); anna.bergamaschi@psi.ch (A.B.); martin.brueckner@psi.ch (M.B.); sabina.chiriotti-alvarez@psi.ch (S.C.); roberto.dinapoli@psi.ch (R.D.); erik.froejdh@psi.ch (E.F.); shqipe.hasanaj@psi.ch (S.H.); pawel.kozlowski@psi.ch (P.K.); carlos.lopez-cuenca@psi.ch (C.L.C.); markus.meyer@psi.ch (M.M.); davide.mezza@psi.ch (D.M.); aldo.mozzanica@psi.ch (A.M.); sophie.redford@psi.ch (S.R.); christian.ruder@psi.ch (C.R.); bernd.schmitt@psi.ch (B.S.); dhanya.thattil@psi.ch (D.T.); gemma.tinti@psi.ch (G.T.); seraphin.vetter@psi.ch (S.V.); jiaguo.zhang@psi.ch (J.Z.); 2 European Synchrotron Radiation Facility (ESRF), 71 Avenue des Martyrs, F-38043 Grenoble, France; paolo.busca@esrf.fr (P.B.); fajardo@esrf.fr (P.F.); marie.ruat@esrf.fr (M.R.); 3 R&D Center “Advanced Electronic Technologies”, Tomsk State University (TSU), Lenin Ave 36, RUS-634050 Tomsk, Russia; ivan_sherbakov94@mail.ru (I.C.); gaas.workshop@gmail.com (A.L.); top@mail.ru (O.T.); antontyazhev@mail.ru (A.T.); zarubin_an@mail.ru (A.Z.)

**Keywords:** GaAs, chromium compensated, JUNGFRAU, crater effect, effective pixel size, pencil beam

## Abstract

Chromium compensated GaAs or GaAs:Cr sensors provided by the Tomsk State University (Russia) were characterized using the low noise, charge integrating readout chip JUNGFRAU with a pixel pitch of 75 × 75 µm^2^ regarding its application as an X-ray detector at synchrotrons sources or FELs. Sensor properties such as dark current, resistivity, noise performance, spectral resolution capability and charge transport properties were measured and compared with results from a previous batch of GaAs:Cr sensors which were produced from wafers obtained from a different supplier. The properties of the sample from the later batch of sensors from 2017 show a resistivity of 1.69 × 10^9^ Ω/cm, which is 47% higher compared to the previous batch from 2016. Moreover, its noise performance is 14% lower with a value of (101.65 ± 0.04) e^−^ ENC and the resolution of a monochromatic 60 keV photo peak is significantly improved by 38% to a FWHM of 4.3%. Likely, this is due to improvements in charge collection, lower noise, and more homogeneous effective pixel size. In a previous work, a hole lifetime of 1.4 ns for GaAs:Cr sensors was determined for the sensors of the 2016 sensor batch, explaining the so-called “crater effect” which describes the occurrence of negative signals in the pixels around a pixel with a photon hit due to the missing hole contribution to the overall signal causing an incomplete signal induction. In this publication, the “crater effect” is further elaborated by measuring GaAs:Cr sensors using the sensors from 2017. The hole lifetime of these sensors was 2.5 ns. A focused photon beam was used to illuminate well defined positions along the pixels in order to corroborate the findings from the previous work and to further characterize the consequences of the “crater effect” on the detector operation.

## 1. Introduction

Semiconductor sensors made of silicon offer outstanding performance in terms of response uniformity and charge transport properties. However, due to the relatively low atomic number Z of silicon (Z_Si_ = 14), its absorption efficiency decreases rapidly for photon energies above 20 keV. One possibility to overcome this limitation is using semiconductor sensors with higher atomic numbers like CdTe (Z_Cd/Te_ = 48/52) or GaAs (Z_Ga/As_ = 31/33) [1]. On the other hand, High-Z sensors, being compound semiconductors, still do not provide the same response uniformity as silicon and suffer from significant auto-fluorescence and charge trapping effects like polarization and afterglow [2,3,4,5].

Initially investigated to be used as semiconductor sensor material in the 1960s, GaAs has experienced a revival as sensor material after researchers from the Tomsk State University (TSU) have presented a way to obtain high resistivity GaAs by diffusing chromium in a post-processing step into low resistivity n-type GaAs sensors that are commercially available [6,7,8]. That allows for the production of GaAs:Cr sensors with a resistivity higher than 10^9^ Ω/cm. Further details on the compensation process are explained in Reference [9]. Chromium compensated GaAs has been successfully evaluated as sensor material by several groups as it exhibits good spectral performance and stable detector operation, even under high photon fluxes which makes it a promising sensor material for X-ray detectors at synchrotrons or FELs [9,10,11,12,13,14,15,16].

In a previous paper, GaAs:Cr sensors grown from TSU grown by the Liquid Encapsulated Czochralski (LEC) technique have been investigated with the low-noise, charge integrating readout chip JUNGFRAU, which enables a direct measurement of charge collected within a small volume of the sensor due to the (relatively) small pixel pitch of 75 × 75 µm^2^ [17]. The wafers were obtained from vendor #1 and the sensors were produced in 2016. One of the key findings of the previous publication was the experimental determination of the hole lifetime in GaAs:Cr sensors of 1.4 ns. This comparably short lifetime allows drift lengths of holes of only few tens of microns within the GaAs:Cr sensor, which can cause an incomplete signal induction in the collecting pixel electrode and a negative signal in the adjacent pixels depending on the point of photon absorption within the pixel. This effect can create pixel clusters with a halo of negative signals around the central pixel which was called “crater effect” and is further investigated in this study.

In this publication, 500 μm thick GaAs:Cr sensors produced by TSU in 2017 from commercially available n-type GaAs wafers grown by LEC, but coming from a different vendor #2, are characterized. GaAs:Cr sensors were bump bonded to JUNGFRAU readout chips to perform a basic sensor characterization where parameters like noise, spectral resolution capability and charge transport properties are measured. These results were compared with the material from vendor #1 which was previously characterized. Further measurements about the origin and the consequences of the crater effect are shown by using a focused photon beam at a synchrotron source to illuminate well defined positions along the pixel depth to experimentally validate the assumptions from the previous paper.

## 2. Readout Chip and Test System

GaAs:Cr sensors, bump bonded at the Paul Scherrer Institut (PSI), were characterized with the low noise, charge-integrating readout chip JUNGFRAU1.0 which has been developed by the Photon Science Detector Group of PSI in Switzerland [18,19]. The pixel array consists of 256 × 256 pixels with a pixel size of 75 × 75 µm^2^. JUNGFRAU1.0 has three gain implemented in the preamplifier stage which are realized by three differently sized capacitors in the preamplifier’s feedback loop. In hole collection mode, the gain range is automatically adapted to the incoming amount of photons/charge, which is not possible in electron collection mode. (At the time of writing this publication, a new version of the readout chip (JUNGFRAU1.1), which is supporting native electron collection, was designed, thus being able to automatically adapt its gain when collecting negative charge. In case of high-Z sensors, where electron collection is preferred due to the superior charge transport properties of electrons, the gain had to be set before each acquisition, based on the expected number of impinging photons. If not explicitly stated otherwise, the highest gain “G0” was used for the measurements.

JUNGFRAU is a charge-integrating readout chip and when operated in fixed gain, data processing includes the following steps:

1. Pedestal correction: Typically, all frames were corrected for an offset, called pedestal, that is mainly defined by the chip settings and the dark current integrated during the pre-defined integration time window. This pedestal is a constant value which is evaluated per pixel either before the measurement by acquiring 5000 dark frames (when a high photon occupancy is expected) or on-the-fly during the experiment in a low photon flux environment. The variation of the pedestal (in r.m.s.) corresponds to the noise of each pixel of the detector system, including contributions from the readout chip and the sensor.
2. Gain correction: The output of the pixel is measured by digitizing the analog information with an off-chip ADC (analog-to-digital converter) to a unit named ADU (analog-to-digital unit). In order to convert ADU into keV, several monochromatic energy spectra were taken, the photo peak positions fitted by a Gaussian function and a pixelwise gain map extracted.

The testbed for the evaluation of single chip sized sensors is a modified standard JUNGFRAU multi-ASIC module, which typically hosts 4 × 2 readout chips bump bonded to a monolithic silicon sensor. The advantages of modifying a standard module are the easy integration for operating at synchrotron beamlines and to make use of the dedicated readout system which enables frame rates of up to 1 kHz. For the measurements reported here, the frame rate was set to 500 Hz.

**Figure 1:**
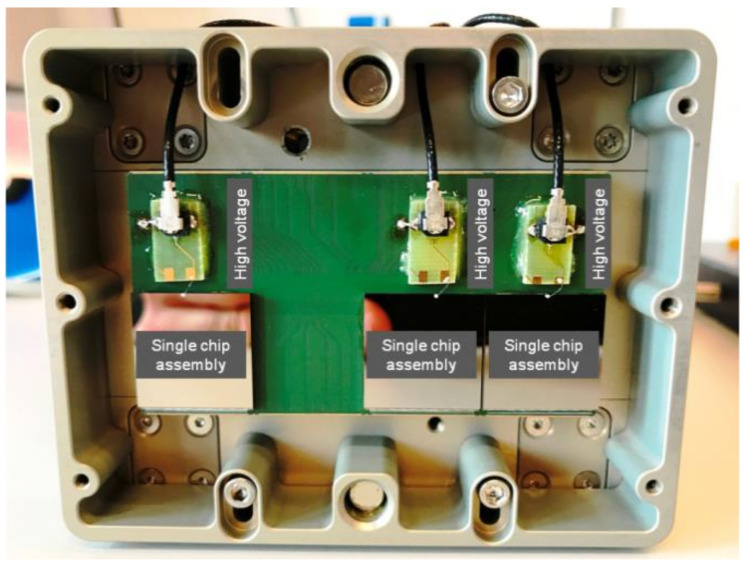
Example of the testbed to evaluate single chip sized sensors connected to JUNGRAU readout chips. The system is based on a modified standard JUNGFRAU multi-ASIC module. In this example three single chip sized hybrid assemblies were mounted. Each hybrid assembly has its own sensor bias voltage connection and can be operated independently.

A modified module is capable of housing up to three different single chip sized hybrid assemblies consisting of JUNGFRAU readout chips in combination with different sensors such as silicon, GaAs or Cd(Zn)Te. The sensor high voltage of each hybrid assembly can be individually provided by external high voltage sources (Figure 1).

The temperature of the housing is actively controlled by using a liquid coolant (a mixture of water and glycol with a freezing point of around −20 °C, the coolant temperature is stabilized within ±0.5 K). Before each measurement, the detector system was operated for around 20 min at the foreseen operating temperature to ensure stable operating conditions during the measurement. Typically, a coolant temperature of T = +15 °C was chosen which allowed an operation of the module without protection measures against condensation.

Moreover, if not explicitly stated otherwise, the GaAs:Cr sensors were biased with a high voltage of USensor = −300 V.

## 3. Results

### 3.1. Dark Current and Dynamic Range

As JUNGFRAU is a charge-integrating readout chip, the pixel output represents the charge collected during a pre-defined integration window. Thus, the dark current of the sensor can be extracted by measuring the integrated charge as a function of the integration time without external stimulus. A linear fit was used to determine the dark current on a pixel-by-pixel basis. The upper fit limit was defined by the integration time value, where the relative deviation between I-t curve and linear fit is larger than 3%. The slope of the linear fit, corresponding to the dark current in units of ADU per µs, was transferred into Ampere using the following conversion:

$$\frac{\mathrm{ADU}}{\mu s}\;\overset{1}{\to }\frac{\mathrm{keV}}{\mu s}\;\overset{2}{\to }\;\frac{n}{\mu s}\;\overset{3}{\to }A$$

1. Conversion by dividing with the pixel gain (ADU/keV) (details about the procedure to obtain the pixel gain can be found in Reference [17]).
2. Conversion into number of charge carriers n by using the electron-hole pair creation energy of 4.2 eV per electron-hole pair [2].
3. Conversion into current by converting into C/s = A, where Q_tot_ = n e.

**Figure 2:**
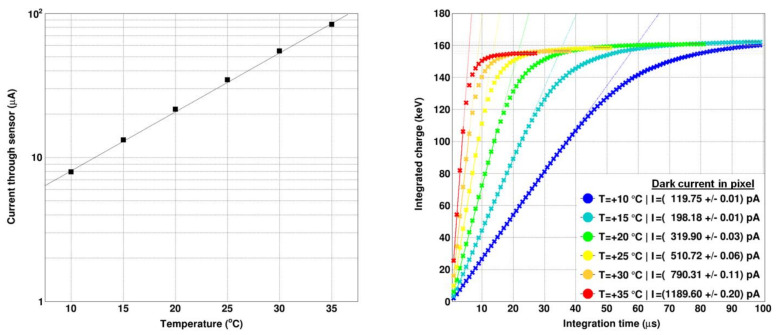
(**left**) Current through the 256 × 256 pixels of the sensor as function of the temperature, showing an exponential increase of the bulk current as function of temperature (**right**) Extracted current from an arbitrary pixel as function of the temperature.

**Figure 3:**
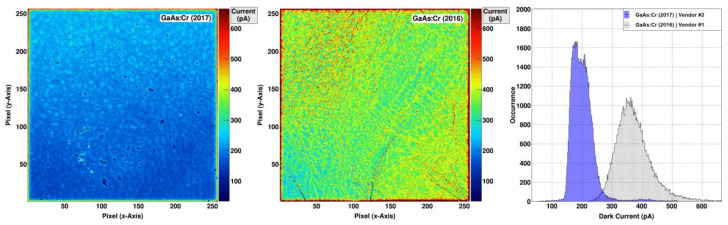
(**left**) Dark current map of the GaAs:Cr (2017) from vendor #2 and (middle) the GaAs:Cr (2016) sensor from vendor #1 measured at a temperature of +15 °C. (**right**) Histogram of the dark currents of both GaAs:Cr sensors. The mean current per pixel is 201.6 pA with a dispersion of 17.3 % (vendor #2) compared to 391.0 pA with a dispersion of 16.9 % (vendor #1).

The dark currents of the sensors were measured in a temperature range between +10 °C and +35 °C. The integral bulk current through all 256 × 256 pixels was calculated by summing up the currents of the single pixels. The total dark current changes from 8.0 µA at T = +10 °C up to 83.7 µA at T = +35 °C (Figure 2, left). At the typical operation conditions with a temperature of T = +15 °C, the dark current through the pixel matrix is 13.38 µA. The mean dark current through a single pixel is (201.6 ± 0.2) pA with a dispersion of 17.3% (r.m.s.) over the pixel matrix (Figure 3, left).

Over the whole pixel matrix, an overall mean dynamic range (until one of the pixel buffers is saturated) of around 160 keV is available per pixel, the mean linear dynamic range (until a deviation of 3% from the linear fit is reached, the same criterion as for the upper fit limit of the dark current) is around 112 keV (with a dispersion of ±3 keV (r.m.s.)). The effect of the sensor temperature on the dark current for an arbitrary single pixel is shown in Figure 2 (right). The dark current fills the linear dynamic range of the pixel at rates between 26.9 keV/µs at T = +35 °C and 2.7 keV/µs at T = +10 °C. Translating that into the time until the linear dynamic range is saturated by the dark current, yields values between 4.1 µs (T =+35 °C) and 41.1 µs (T =+10 °C).

For all the following measurements, a standard operation temperature of +15 °C was chosen and a default integration time of t_int_ = 5 µs defined. By using these settings, around 22.4 keV of dynamic range is filled with dark current (at a rate of 4.5 keV/µs), leaving a linear dynamic range of around 87.1 keV.

A comparison between the dark current maps of the GaAs sensors from vendor #1 (2016) and #2 (2017) is shown in Figure 3 (left, middle). Using the standard operation parameters, the dark current through the bulk from vendor #2 of 13.38 µA is 47% less than through the material from vendor #1 (25.12 µA). As JUNGFRAU is a charge-integrating readout chip and no dark current compensation is available, such a reduction of the sensors dark current is highly appreciated as it allows longer integration times (or less aggressive cooling).

The dark current maps of both GaAs:Cr sensors show relatively strong variations of the dark current between the pixels. However, as the integrated dark current of each pixel is stable in time, pedestal corrections can be applied.

Although a guard ring is used in each sensor, the currents through the edge pixels are significantly increased (more than 100%) compared to the mean values.

By calculating the resistivity using the values of the mean dark current per pixel, a resistiviy of the GaAs:Cr (2017) sensor of 1.69 × 10^9^ Ω/cm (vendor #2) with a dispersion of 17.3% (r.m.s.) over the pixel matrix can be obtained. The resistivity of the material from vendor #1 was 0.85 × 10^9^ Ω/cm with a dispersion of 15.1% (r.m.s.) [17]. These values are well within the range reported by other groups [9,20,21].

### 3.2. Noise and Spectral Capabilities

The noise performance was determined by measuring the pixel output variation using dark frames, i.e., without external stimulus like photons. For each pixel, the output was sampled several thousand times, fed into a histogram and fitted with a Gaussian function. The root mean square (r.m.s.) of this distribution yielded the noise in units of ADU and was subsequently converted into e^−^ ENC (=Equivalent Noise Charge, measured in electrons r.m.s.).

**Figure 4:**
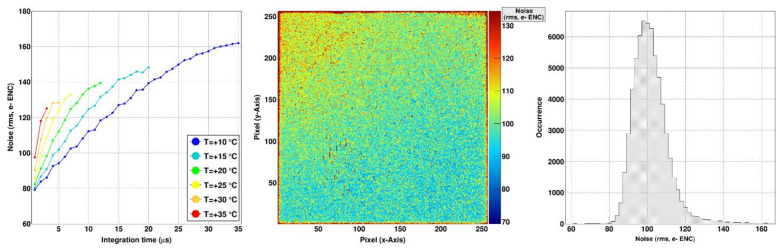
(**left**) Noise performance of the GaAs:Cr (2017) sensor biased at –300 V for various integration times and temperatures. (**middle**/**right**) Map and distribution of the noise performance at the typical opera-tion parameters (T = +15 °C and tint= 5 µs). The mean noise performance is (101.65 ± 0.04) e- ENC with a dis-persion of 9.4%.

The mean noise performance of the GaAs:Cr (2017) sensors (U_Sensor_ = −300 V, T = +15 °C, t_int_ = 5 µs) is (101.65 ± 0.04) e^−^ ENC or 0.427 keV with a dispersion of 9.4% (Figure 4). As the noise depends on the dark current, the spatial distribution of the noise follows the same trend as the distribution of the dark currents having higher values in the upper half of the sensor (Figure 3, left). The noise increases with longer integration times, most likely dominated by the increased shot noise coming from the dark current. In comparison, the noise performance of the previously characterized GaAs:Cr (2016) sensors with the same settings is 14% worse with a mean noise value of (115.93 ± 0.03) e^−^ ENC (or 0.487 keV) and a dispersion of 5.5%.

Caused by the relatively high dark currents, the noise performance of the test systems with GaAs:Cr sensors is significantly worse compared to the noise of assemblies with silicon sensors (83 e^−^ ENC or 0.300 keV) [19]. However, the noise performance is still at an acceptable level.

The spectral performance of the GaAs:Cr sensors was measured by illuminating the assembly with monochromatic 60 keV photons at the BM05 beamline of the ESRF [22]. The assembly was irradiated with a photon flux of around 2 × 10^5^ ph/(mm^2^/s) which was low enough to ensure the detection of isolated photons. A cluster finding algorithm was used (on pedestal und gain corrected data) to identify the pixel clusters, where a photon has been absorbed. The criterion to define a photon hit is the signal in a pixel being higher than 10 × its noise value. The cluster finder algorithm searches in the adjacent pixels, if there is a higher signal present and centers the resulting 3 × 3 pixel cluster in the pixel with the highest signal using an iterative approach (which terminates after 5 iterations due to a likely overlap of photon clusters) [17]. The three outermost pixel rows/columns are not scanned with the cluster finder, as a full signal reconstruction would not be possible. By summing up signals from adjacent pixels, charge which was collected in neighboring pixels (e.g., due to charge sharing or due to fluorescence photons) can be recovered.

**Figure 5:**
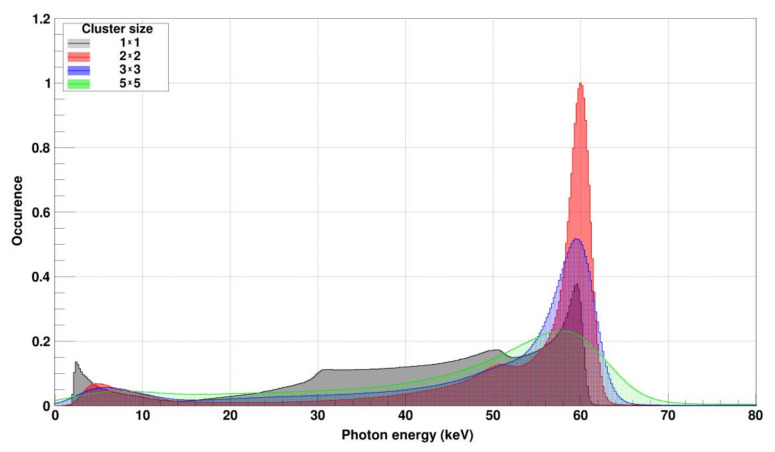
Energy spectrum of 60 keV monochromatic photons measured with a GaAs:Cr sensor (2017) bump bonded to a JUNGFRAU readout chip. A cluster finder was used to identify photons and to center the cluster around the pixel with the highest signal. The gray spectrum uses only the information from the cen-tral pixel of the cluster (1 × 1), e.g., the pixel with the highest signal. Escape photons as well as a relatively high charge sharing tail are visible. The red spectrum is composed of the four pixels of the pixel cluster with the highest summed signal (2 × 2 cluster). In this case, the FWHM for the whole pixel matrix is 2.58 keV or 4.3%. The blue spectrum uses the summed signal from the central pixel plus its eight neighbors (3 × 3 clus-ter), the green spectrum the information from a 5 × 5 pixel cluster centered around the pixel with the highest signal. (The increase of the photo peak width with bigger clusters is due to the higher number of pixels and thus their summed up noise contributions using clustering).

The FWHM of the 60 keV peak from the spectrum obtained by the summed signal from 2 × 2 pixel clusters is 2.58 keV i.e., an energy resolution of 4.3% for the whole pixel matrix for the GaAs:Cr sensors produced in 2017 (Figure 5). The corresponding measurement with the GaAs:Cr 2016 sensor yielded a FHWM of 4.14 keV i.e., an energy resolution of 6.9%. These results are in agreement with results measured by other authors [9].

### 3.3. Charge Transport Properties

The charge carrier transport properties, namely the mobility-lifetime or *μ·τ* product, of the GaAs:Cr (2017) sensors have been determined, as the *μ·τ* product plays a key role in the signal induction process. It was determined using the Hecht relationship which describes the induced signal of charge carriers as a function of the applied high voltage of the sensor [23,24]. In order to measure the *µ·τ* product of either holes or electrons, fluorescence photons of molybdenum with an energy of 17.4 keV have been used, which deposit a well-defined amount of charge within the first few tens of microns of the sensor. The Hecht relationship modified by Hamann to take into account charge trapping and the small pixel effect (i.e., a position dependence of the signal induction, where most of the signal is induced close to the collecting electrode) has been used for fitting the data [2,15,19,20] (Equation (1)).

Equation (1): Modified Hecht relationship. (*Q_0_*: Initially deposited charge, *d*: Sensor thickness, *D*: Thickness of sensor, where charge is induced (approximated as pixel pitch in depth), *U*: Sensor bias voltage, *U_0_*: Minimum voltage to induce signal).

(1) $$Q\left(U\right)=Q_{0}\cdot \underset{\mathrm{Charge}\;\mathrm{traping}}{\underbrace{\left[\exp\left(-\frac{\left(d-D\right)\cdot d}{\mu \cdot \tau \cdot (U-U_{0})}\right)\right]}}\cdot \underset{\mathrm{Signal}\;\mathrm{induction}}{\underbrace{\left[\frac{\mu \cdot \tau \cdot (U-U_{0})}{D\cdot d}\cdot \left[1-\exp\left(-\frac{D\cdot d}{\mu \cdot \tau \cdot (U-U_{0})}\right)\right]\right]}}$$

**Figure 6:**
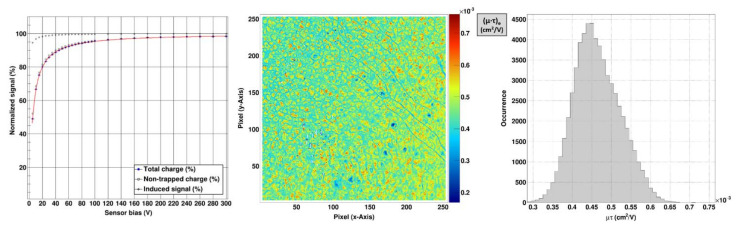
(**left**) Hecht curve of the GaAs:Cr (2017) sensor using molybdenum fluorescence photons with an energy of 17.4 keV. The data was fitted with equation 1 (red solid line). The mean (μ·τ)_e_ is (4.730 ± 0.003) × 10^–4^ cm^2^/V and the charge collection efficiency (CCE) at –300 V is 98.2%. (**middle**) Spatial distribution and (**right**) histogram of (μ·τ)_e_ product of the whole sensor: From the distribution of (μ·τ)_e_ values, extracted on a pixel-by-pixel basis, a mean value of (4.640 ± 0.002) × 10^–4^ cm^2^/V with a dispersion of 12.7%. (The white squares in the map indicate pixel clusters which were masked due to too high noise and/or a pedestal value beyond limit).

The dependence of the signal (normalized to *Q_0_*) induced by electrons as function of the applied sensor voltage is shown in Figure 6 (left). The *µ·τ* product of the GaAs:Cr (2017) sensor for electrons is (4.730 ± 0.003) × 10^−4^ cm^2^/V. The charge collection efficiency (CCE) at the maximum absolute sensor high voltage of −300 V is 98.2%. The spatial distribution in Figure 6 (middle) reveals that the (*µ·τ*) _e_ product along the dislocation lines (blue line structures) is roughly 25% less than the mean value. The dispersion over the pixel matrix is 12.7% (Figure 6, right). Moreover, areas with significantly lower/higher (*µ·τ*) _e_ values resemble structures found in the dark current map (compare Figure 3, left). When reverting the sensor high voltage, no hole signal was visible, probably due to the low hole lifetime in chromium compensated GaAs [17].

For the GaAs:Cr (2016) sensors from vendor #1 the mean *µ·τ* product of electrons was determined to be (1.831 ± 0.002) × 10^−4^ cm^2^/V and the charge collection efficiency at −300 V was 96.0% [17].

### 3.4. Crater Effect

The so-called “crater effect” describes an effect which causes a reduced signal in the collecting pixel and a negative signal in the adjacent pixels due to the short lifetime of holes in chromium compensated GaAs which prevents the holes to (significantly) contribute to the signal induction. The crater effect was already described in a previous publication about LEC-grown GaAs:Cr (2016) sensors [17].

**Figure 7:**
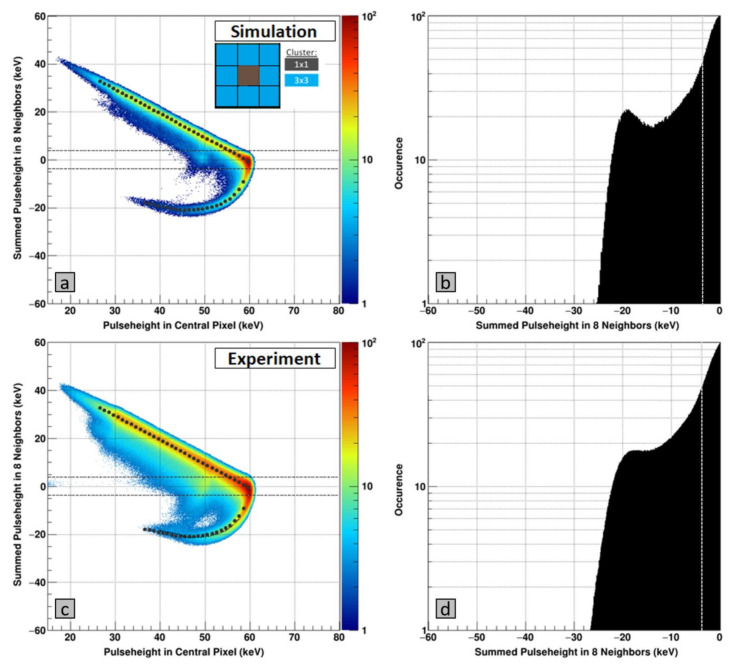
(**left**) Correlation plots of monochromatic 60 keV photons showing the sum of the signals in the 8 adjacent pixels (y-axis) as function of the signal in the central pixel (x-axis) of a 3 × 3 cluster. The horizontal black dotted lines represent the selection criteria of ±3 × the noise of the eight neighboring pixels. (**a**) Correlation plot generated from a simulated dataset using the following parameters: τ_e_ = 120.0 ns, μ_e_ = 4000 cm^2^/(V·s), τ_h_ = 2.5 ns, μ_h_ = 200 cm^2^/(V·s). The gray points indicate the fitted value of the summed pulse height in the eight neighboring pixels. (**c**) Correlation plot generated from experimental data. The gray points indicate the fitted value of the summed pulse height in the eight neighboring pixels for the simulated dataset. (**right**) Projection of the negative signal tail of the correlation plots. The gray vertical line indicates –3× the noise of the eight neighboring pixels, i.e., all events left of this line are considered as crater hits (**b**) Simulation and (**d**) Experimental data.

It can be visualized by means of correlation plots, where the signal in eight outer pixels (*y*-axis) of a 3 × 3 cluster is drawn as function of the signal in the central pixel of the cluster (*x*-axis). An example with experimental data and 60 keV monochromatic photons is shown for the GaAs:Cr (2017) sensor in Figure 7 (bottom left). By categorizing each event according to the summed signal of the eight neighboring pixels, around 19.6% of all incoming events show a signal which is more negative than 3× the noise of the eight adjacent pixels (being the criterion for a crater hit) (Figure 7, bottom right).

The experimental data were reproduced by simulating the signal induction considering the following processes: X-ray photon absorption (including fluorescence photons) and carrier generation, drift and diffusion (DD) of electrons and holes with constant diffusion coefficients, charge trapping as well as signal formation at the readout electrodes using a calculated weighting potential/field, based on W. Riegler [25]. White noise, corresponding to the noise performance of the JUNGFRAU/GaAs:Cr hybrid assembly with the respective operation parameters, has been added to the resulting signal. Simulations with different combinations of charge carrier mobility and lifetime (for holes and electrons) have been performed. For the electrons, the boundary condition was that the product of mobility and lifetime corresponds to the value obtained by the Hecht measurement within a limit of ±20%.

The best agreement between simulation and experiment is obtained for the charge carrier properties given in Table 1 and shown in the upper plot in Figure 7. The charge carrier properties of the GaAs:Cr (2017) sensor are significantly improved compared to the GaAs:Cr sensor from 2016 (μ_e_ = 2585 cm^2^/(V·s), τ_e_ = 80 ns, μ_h_ = 171 cm^2^/(V·s), τ_h_ = 1.4 ns) [17].

**Figure 8:**
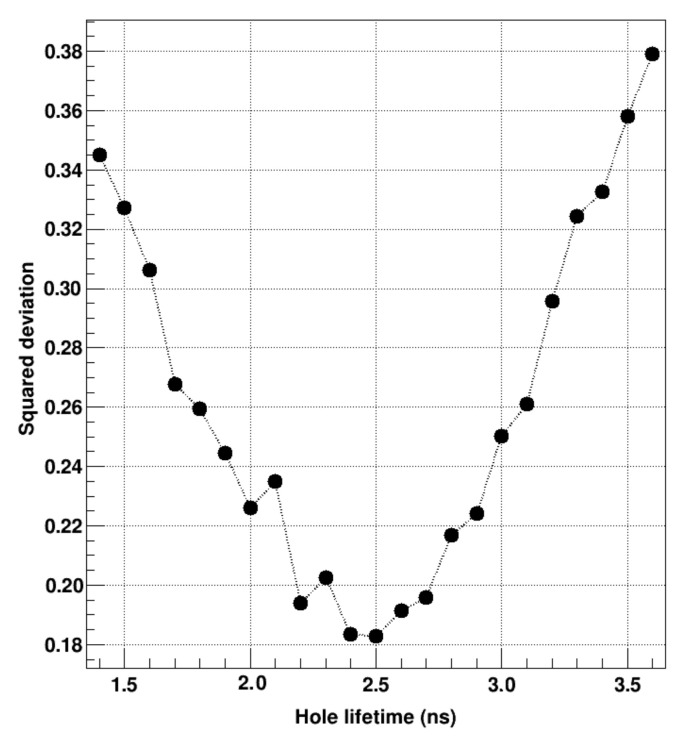
Squared deviation between experimental and simulated data of the negative signal tail of the correlation plots as a function of the hole lifetime.

As the simulations reveal, the negative tail of the correlation plot reacts sensitively to variations of the hole lifetime, while keeping the hole mobility at values typically reported for this material [8,26]. Therefore, the negative tails of the correlation plots were further investigated to determine the best agreement between experimental and simulated data by fitting the values of the summed pulse heights in the eight neighboring pixels as function of the energy in the central pixel using Gaussian fits (Figure 7, left). Subsequently, the difference between experimental and simulated data was calculated at each point. Figure 8 shows the sums of the squared deviations for simulated datasets with different hole lifetimes to give an understanding of the precision in the determination of the hole lifetime.

**Figure 9:**
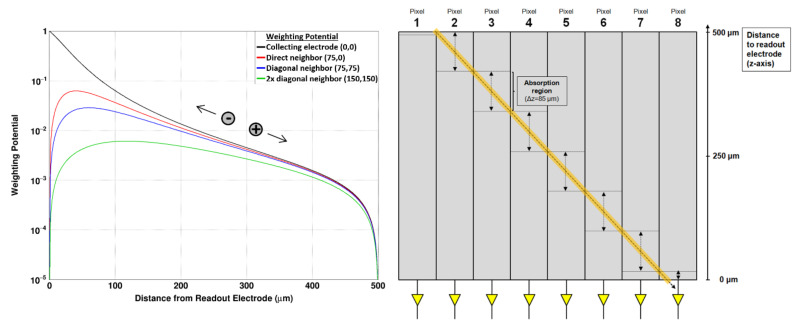
(**left**) Weighting potential calculated according to Riegler et al. at four different positions: above the readout electrode (0 μm, 0 μm), above a direct neighboring pixel (75 μm, 0 μm), above a diagonal neighbor (75 μm, 75 μm), above a diagonal neighbor at twice the distance (150 μm, 150 μm). (**right**) Schematics on the angle-on measurement with a 45 keV focused beam (beam diameter of 10 µm). The penetration angle was 48.6 degrees (with respect to the sensor surface) and the focused beam has been aligned along a pixel row. Overall, eight pixels along a column responded and the photons have been absorbed at different sensor depths. Determined by the penetration angle, the maximum absorption length along the pixel depth Δz in a single pixel was 85 µm.

The reason for the presence of the crater effect is the missing contribution of the hole signal to the overall induced signal. While the generated electrons typically drift through the whole sensor volume, the drift length of the generated holes with a lifetime of 2.5 ns and a hole mobility of 200 cm^2^/(V·s) is only around 30 µm (for a typical sensor bias voltage U_Sensor_ = −300 V and a sensor thickness of d = 500 µm). Taking into account the Shockley-Ramo theorem [4], this has two consequences:

1. Collecting electrode: For photons that are absorbed close to the readout electrode (few to several tens of microns), although there is the small pixel effect, the electrons induce only a fraction of the overall signal. Due to the relatively short drift length of the holes (compared to the sensor thickness), the hole contribution is not enough to induce the full signal (Figure 9, left—black line).
2. Neighboring electrode(s): When a photon is absorbed in the proximity of the readout electrode, the electrons that drift to the collecting electrode induce a negative signal in the adjacent pixels (Figure 9, left—red/blue/green line). The holes compensate for this signal, while moving towards the backside contact. However, due to the limited drift length of the holes, the hole contribution is not sufficient to cancel the negative signal and the overall induced signal remains negative.

#### 3.4.1. Angle-On Measurement

In order to further investigate the crater effect, measurements with a pencil beam tilted by 48.6 degrees (with respect to the sensor surface) have been performed at the BM05 beamline of the ESRF. The spot diameter of the photon beam was between 5 and 10 µm. The experiments were performed at a low photon flux with a monochromatic photon energy of 45 keV. As the absorption length (1/e) of photons with an energy of 45 keV is 417 µm in GaAs, photons will be absorbed along the whole distance of roughly 667 µm through the 500 μm thick sensor. Effectively, photons will be absorbed at a (relatively) well-defined depth depending on the responding pixel. The focused beam was aligned in parallel along a pixel row, so that eight pixels showed a response along one column (Figure 9, right). Due to the penetration angle, the typical interaction range along the *z*-axis was different for each pixel, covering a photon absorption range of Δz = 85 µm for each pixel (except the most outside pixels 1, 2 and 8).

The distances to the collecting electrode of each pixel are summarized in Table 2. Moreover, from the estimated charge transport values for the GaAs:Cr sensors summarized in Table 1, the induced signal in the collecting electrode as well as the sum of induced signals in the eight adjacent neighbors can be calculated using the Shockley–Ramo theorem and the weighing potential from Figure 9 (left).

**Figure 10:**
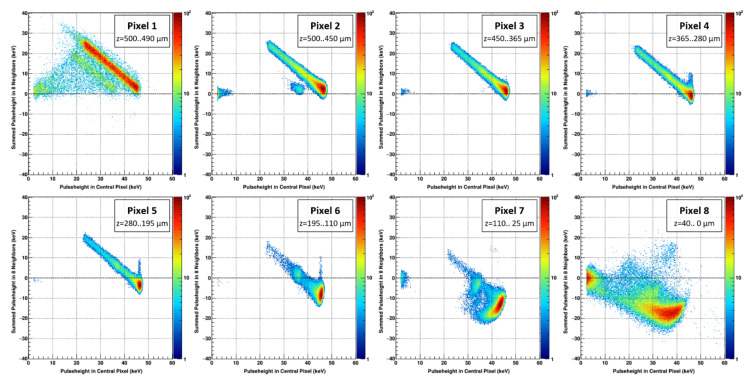
Experimental data of the 45 keV focused beam impinging on the sensor at a 48.6 degree angle. The plots show the correlation between the sum of the signals in the eight neighbors (y-axis) as function of the signal in the central pixel (x-axis) of a 3 × 3 cluster for different absorption depths in the sensor. The z-axis of each plot is normalized to its highest signal.

The previously mentioned cluster finding algorithm was employed to ensure that only frames with a single photon hit were used for the data analysis. Each pixel cluster was assigned to the pixel with the highest signal and correlation plots of the sum of the signals in the eight adjacent pixels (*y*-axis) as function of the signal in the central pixel (*x*-axis) have been generated for the different interaction points within the sensor (Figure 10).

**Figure 11:**
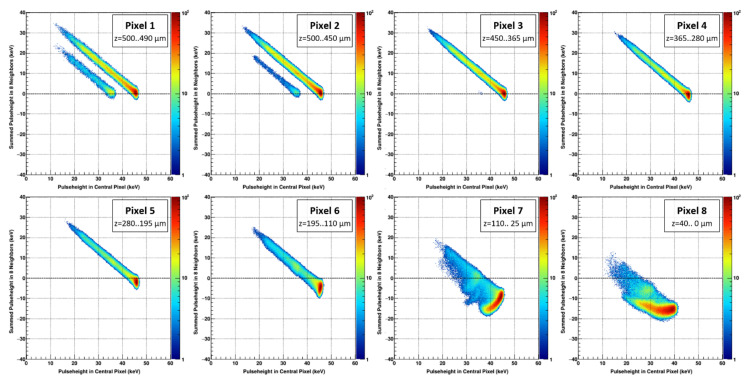
Simulated data of 45 keV photons absorbed at different absorption depths of the sensor (according to the depth bins of the experimental data in Figure 10).

In order to simulate the experimental findings, datasets using the same beam parameters were created. The absorption of 45 keV photons at random positions within the sensor (using the previously estimated charge transport properties from Table 1) were treated with the same analysis script which was used for the analysis of the measured data. The simulated interactions have been assigned to eight virtual pixels corresponding to the absorption depth range of the respective pixels from the experiment. The correlation plots, simulating photon absorption in a specific layer, were used to reproduce the experimental data (Figure 11).

**Figure 12:**
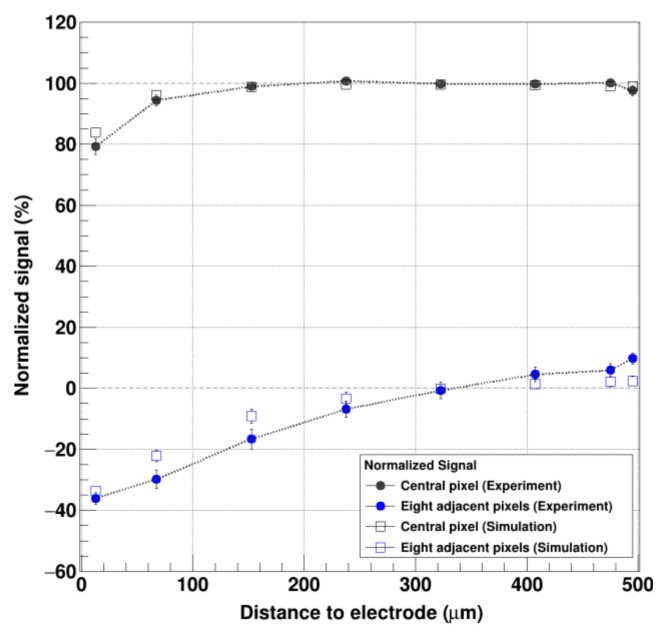
Comparison of the experimental and simulated data of the angle-on measurement: (Black solid indicator: experimental data/black square: simulated data) Signal in the central pixel of a 3 × 3 cluster as function of the absorption distance from the electrode. (Blue solid indicator: experimental data/blue square: simulated data) Signal in the eight adjacent pixels.

The simulated data are shown in Figure 11 and agree well with the experimental data from Figure 10. The experimental and simulated data with the previously obtained parameters are able to illustrate the consequences of the short hole lifetime, namely the reduced photo peak signal and a more negative signal in the adjacent pixels the closer the photons are absorbed to the readout electrode. Moreover, the results from the angle-on measurement are in good agreement with the analytical calculations using the Shockley–Ramo theorem (Table 2/Figure 12).

The consequences of the incomplete signal induction due to the missing hole contribution can be described for pixels 1–8 as follows (Figure 12):

- Absorption in the first half of the sensor/Distance to the readout electrode = 250–500 µm: The absorption of the photons in case of pixels 1–4 happens in the first half of the sensor, i.e., the vast majority of the signal in the collecting electrode is induced by the electrons due to the small pixel effect. A photo peak in the central pixel is clearly visible at 45 keV. The diagonal line spanning from the full signal in the central pixel at 45 keV indicates events where the charge is shared with neighboring pixels. The shallower the photon absorption (Pixel 4→1), the higher the signal in the neighboring pixels at the position of the photo peak, indicating more charge sharing due to the longer drift length (Figure 10 and Figure 11). Additionally, in the case of pixels 1 and 2, escape peaks are visible, likely due to fluorescence photons that escaped through the entrance side of the sensor. Due to the limited range of the fluorescence photons, no escape peaks appear in the pixels 3 and 4. (Please note that pixel 1 was only hit close to the border of pixel 2 which explains the rather low statistics and the high amount of charge sharing).
- Absorption in the second half of the sensor/Distance to the readout electrode = 0–250 µm: The signals from the photons registered in pixel 5–8 are induced both by electrons drifting to the pixel electrode as well as holes drifting to the backside contact (the amount depends on the distance to the pixel electrode) (Figure 9, left). The signal in the central pixel decreases from (100.7 ± 0.8)% in pixel 5 to a broad peak at around (79.2 ± 2.6)% in pixel 8. The sum of the induced signals in the eight neighboring pixels also gets more negative as the absorption occurs closer to the readout electrode from (−6.9 ± 2.7)% at pixel 5 to (−36.1 ± 1.9)% in pixel 8. Notably, the spectra of the pixels 7 and 8 show escape peaks again, in this case likely by escaped fluorescence photons through the ASIC.

#### 3.4.2. Edge-On Measurement

**Figure 13:**
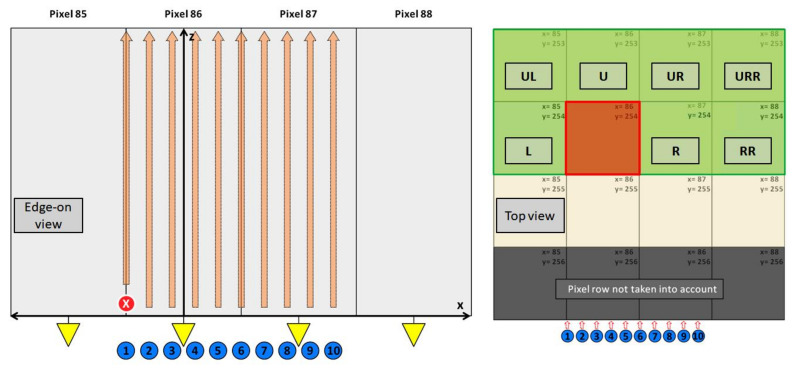
(**left**) Diagram of the edge-on measurement. The pixel rows 86 and 87 have been raster scanned with a focused 40 keV photon beam with a granularity of Δx = 15 µm (10 scan points) and a Δz = 10 µm (60 scan points). (**right**) The plot shows the top view on 4 × 4 pixels (x = 85–88 and y = 253–256). By using a photon finder algorithm only photons that were absorbed in the pixel row 254 were taken into account to avoid sensor corner effect. The response maps of the adjacent pixels were created, indicating the response of a pixel depending on the interaction point in the pixel under investigation (86, 254). As example, the plot indicates the assignment, when the photon absorption happens in pixel (86, 254) (red square). The letters indicate the following pixels (w.r.t. the pixel under investigation): L—left pixel, R—right pixel, RR—2 × right pixel, UL—upper left pixel, U—upper pixel, UR—upper right pixel, URR—upper 2 × right pixel. During the experiment two pixels have been scanned, namely (86, 254) and (87, 254).

In order to investigate the signal induction along the depth of the sensor in more detail, the sensor edge has been placed perpendicular to the focused photon beam and an edge-on raster scan was performed. The photon energy was 40 keV and the focal spot size was 5–10 µm. The photon beam was scanned along the side of the sensor at an angle of 90° to probe how the measured signal changes depending on the absorption position in the sensor (Figure 13, left). The step size along the sensor depth was Δz = 10 µm and Δx = 15 µm along the *x*-axis. In order to better define the absorption in the y-direction, i.e., in beam direction, and to minimize sensor corner effects, a cluster finder algorithm was employed to use only events that were absorbed in the central region (approximately Δy = ±20 µm) of two pixels (x = 86 y = 254 and x = 87 y = 254) (Figure 13, right) and the pedestal was updated for each raster scan point.

**Figure 14:**
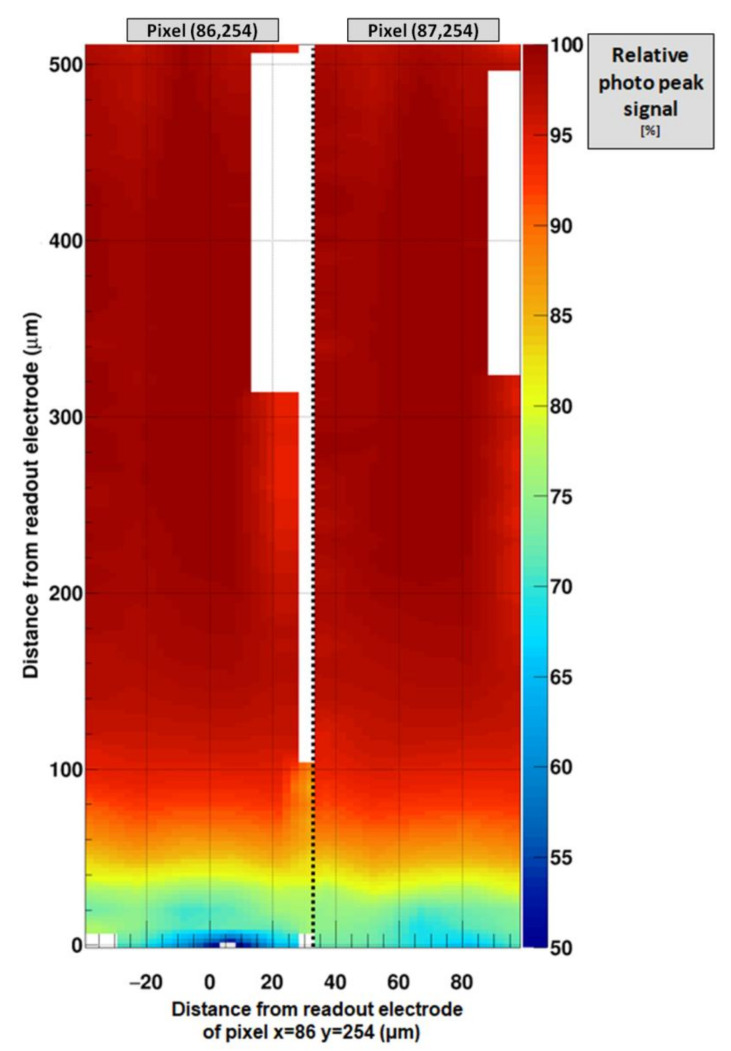
Relative signal of the photo peak for the two pixels under investigation: x = 0 indicates the center of pixel (86, 254), x = 75 indicates the center of pixel (87, 254), the black dashed line indicates the pixel boundary. The reduction of the induced signal due to the missing hole contribution, when the photons are absorbed close to the readout electrode, is clearly visible. For the white areas, no photo peak could be estimated reliably due to too much charge sharing. It is unclear whether the unsymmetrical behavior at the pixel boundary is due to a non-optimal alignment of the scan axis or due to variations of the effective pixel size in GaAs:Cr sensors (see following chapter).

From these edge-on measurements several sensor characteristics can be revealed:

- Charge collection efficiency (CCE):

The photo peak signal was extracted for each interaction point of the edge-on scan. The photo peak was fitted by a Gaussian function and the obtained signal (in keV) normalized to the incoming photon energy.In agreement with the angle-on measurement, the charge collection efficiency (CCE) is decreasing to values of around 65%, the closer photons are absorbed to the readout electrode (Figure 14).

- Crater probability and signal height:

The criterion for a “crater” was previously defined as the sum of the signals of the eight adjacent pixels around the central pixel of a 3 × 3 cluster (centered around the pixel with the highest signal) being more negative than −3× the summed noise of these eight pixels. For the edge-on scans, as the response of single pixels is investigated, a “crater” is considered to be present, when the signal of a single pixel is more negative than −3× the noise of the respective pixel.

**Figure 15:**
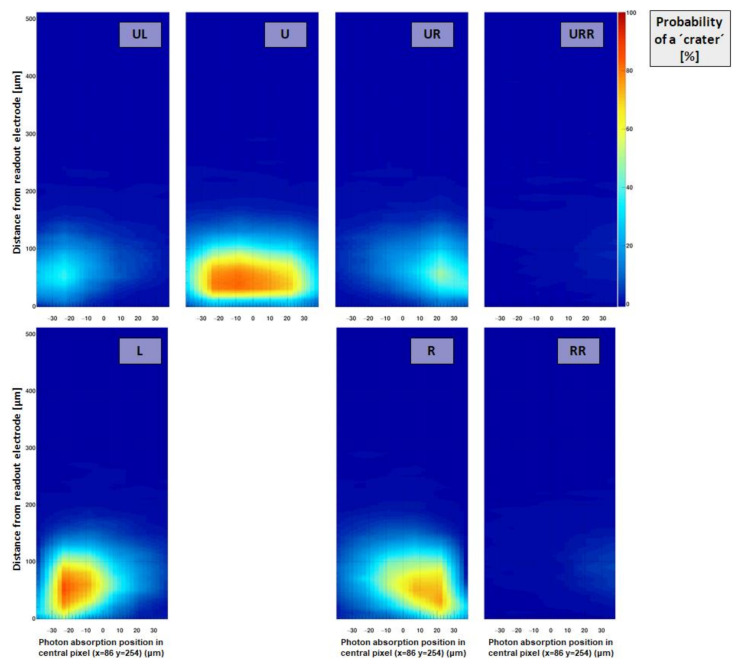
Relative number (or “crater probability”) of hits causing a strongly negative signal (<−5 × pixel noise) depending on the interaction point in the pixel under investigation for different pixels in the direct neighborhood. The directly adjacent pixels (R/U/L) exhibit high probabilities close to 100%, when photons are deposited close to the readout electrode. The crater probability drops close to the pixel boundaries (at ±37.5 μm) due to enhanced charge sharing. Note: Compared to Figure 14, where the pixel response is shown as function of absorption position in the pixel itself, this plot indicates the pixel response of a pixel in the vicinity of the irradiated pixel as function of the absorption position within the pixel which is being irradiated.

**Figure 16:**
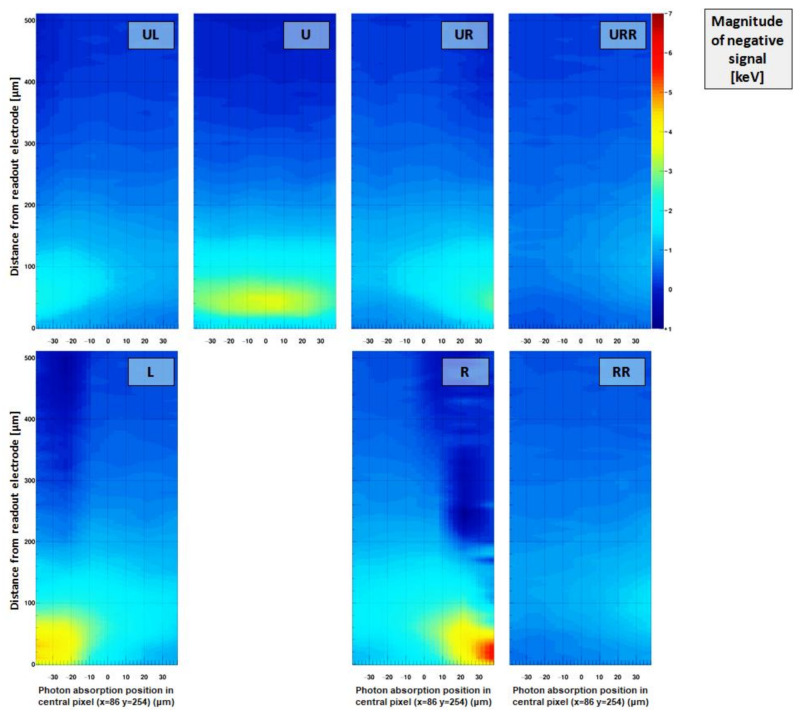
Intensity of the negative signal induced in pixels surrounding the pixel under investigation. The direct neighboring pixels (L/U/R) show negative signals which on average reach around –10% or –4 keV of the incoming photon energy of 40 keV. The diagonal neighboring pixels (UL/UR) still exhibit negative signals down to around –5% or –2 keV. Even pixels further distant (RR/URR) show slightly negative mean values. It is obvious that the negative signal in the adjacent pixels is caused by photons absorbed close to the readout electrode, validating the assumptions previously made in the paper. Please note: Compared to Figure 14, where the pixel response is shown as function of the absorption position in the pixel itself, this plot indicates the pixel response of a pixel in the vicinity of the irradiated pixel as function of the absorption position within the pixel which is being irradiated.

Figure 15 shows the probability of observing a strongly negative signal (i.e., a “crater”) in neighboring pixels and Figure 16 reveals the magnitude of the negative signal in neighboring pixels, depending on the absorption position of a photon within the pixel under investigation (x = 86 y = 254). The probability was calculated by dividing the number of crater events by the overall number of absorbed photons for each raster step.

- Direct neighbors (L/U/R):

For pixels directly adjacent to the absorbing pixel, namely L/R, the probability to observe a signal less than 3 × the noise is between 70 and 100% when the absorption occurs in an area roughly 100 µm away from the pixel (the probability decreases when the photon beam reaches close to the neighboring pixel due to the finite width of the focal spot and charge sharing). The signal in this region close to the readout electrode reaches values of around −4 keV or −10% of the incoming photon energy. The maximum crater probability and negativity of the signal are reached at a distance of around 50 µm or 40 µm away from the readout electrode in z-direction, respectively, which is in good agreement with the predictions given by the weighting potential in Figure 9 (left). The values of crater probability and magnitude are reduced in the directly adjacent pixel U (compared to L/R), as the cluster finder algorithm suppresses photon hits where the photons were absorbed close to the upper pixel boundary (towards the pixel U).

- Diagonal neighbors (UL/UR):

The diagonally adjacent pixels (UL and UR) exhibit a maximum probability of yielding a crater of around 40% roughly 50 µm away from the readout electrodes in z-direction. The negative signal typically reaches values around −1.5 keV or less than 4%.

- Neighboring pixels further away (URR/RR):

The probability (and magnitude) of inducing a negative signal is strongly decreasing with increasing distance to the pixel under investigation. The probability to observe a crater hit is reduced to values below 10–20% and its magnitude lies close to/within the noise limit.

**Figure 17:**
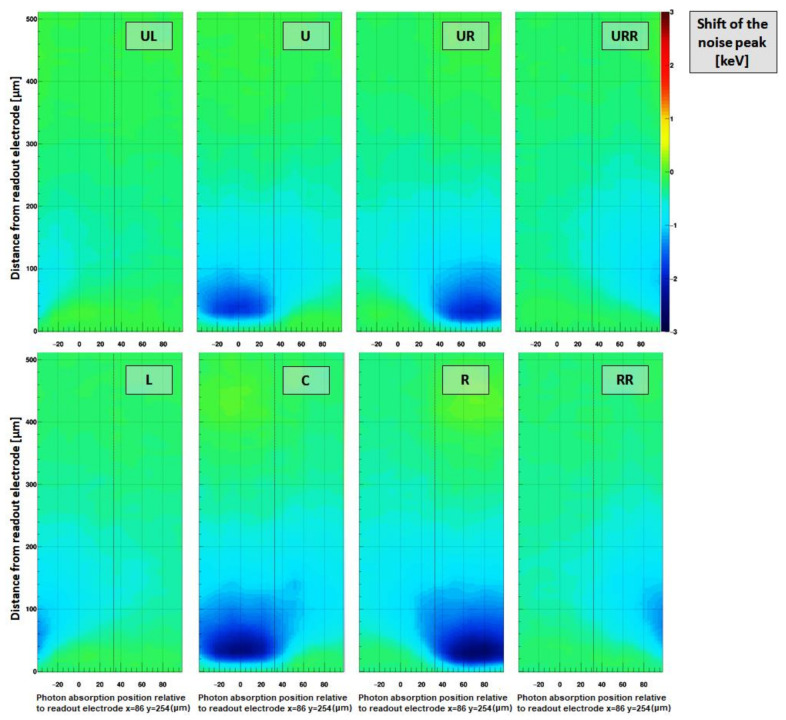
Shift of the pedestal in frames without photons. The irradiated pixels are pixel C (x = 86 y = 254) and pixel R (x = 87 y = 254). The energy position of the pedestal of the eight previously observed pixels is plotted as function of the interaction point in the irradiated pixels. Only frames without photon hits were used for this analysis. The pedestal shift is present, when photons are absorbed in the half of the sensor closer to the readout electrode and it can reach values down to –2 keV. No relaxation effect could be observed, i.e., this pedestal shift has some temporal persistence which has not been further characterized.

- Pedestal shift:

By fitting the pedestal position of frames without photon events, a negative shift of the pedestal can be observed, when photons have been absorbed close to the readout electrode before (Figure 17). The magnitude of this pedestal shift can reach up to −2 keV and is present in frames without photon interaction, i.e., it is a (partially) persistent effect. The origin of the effect is unclear; however, a similar effect has been observed previously also by other authors [13].

### 3.5. Effective Pixel Size

A pencil beam scan was performed at the BM05 beamline of the ESRF to probe the pixel response of single pixels by illuminating a region of interest with a 20 keV photon beam oriented perpendicular to the detector. Due to the low photon energy and thus the shallow, relatively well-defined absorption profile, the generated charge carriers drift through the whole sensor volume to reach the readout electrode. The spot diameter was approximately 5 µm and the beam was raster scanned over a region of interest of the sensor with a step size of 5 µm.

**Figure 18:**
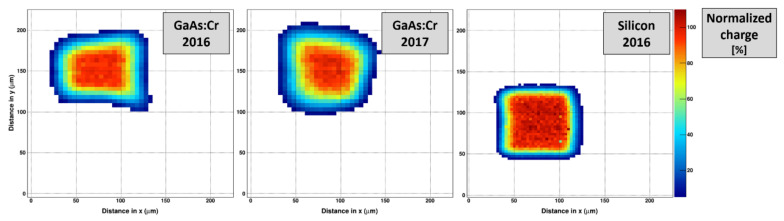
Collected charge in a single pixel as function of the position in x-y direction of a 20 keV pencil beam raster scanned with a step size of 5 µm for three different sensors: (**left**) GaAs:Cr 2016 (**center**) GaAs:Cr 2017 (**right**) Silicon. The z-axis indicates the normalized integrated charge. The minimum of the z-axis is fixed to 5% for a better comprehensibility as it removes contributions from scattered photons and auto fluorescence.

Figure 18 shows examples of the pixel response of single pixels of different sensors as function of the position of the pencil beam. At each scan position in the region of interest (ROI), the response of all pixel in the ROI was recorded. For each pixel, all signals above a threshold of 2.0 keV were summed up and a pixel reponse map for each pixel was created. Compared to the ideal pixel shape that can be obtained with a silicon sensor (Figure 18, right), both GaAs:Cr sensors show significant deviations (Figure 18, left/center). (Possible problems with the experimental setup, e.g., motor movement, can be excluded as the scan with the silicon sensor was performed together with the scan of the GaAs:Cr 2016 sensor).

The single pixel response maps were merged to reveal a potential lateral charge movement or charge loss (e.g., due to recombination centers) in the region of interest. The scan region of the GaAs:Cr 2016 sensors comprises an area of 375 × 375 μm², i.e., fully covering 4 × 4 pixels. For the GaAs:Cr 2017 sensors the scan covers an area of 225 × 225 μm² or 2 × 2 pixels. Unfortunately, the pixels scanned do not coincide with the pixels previously investigated in the edge-on scan.

**Figure 19:**
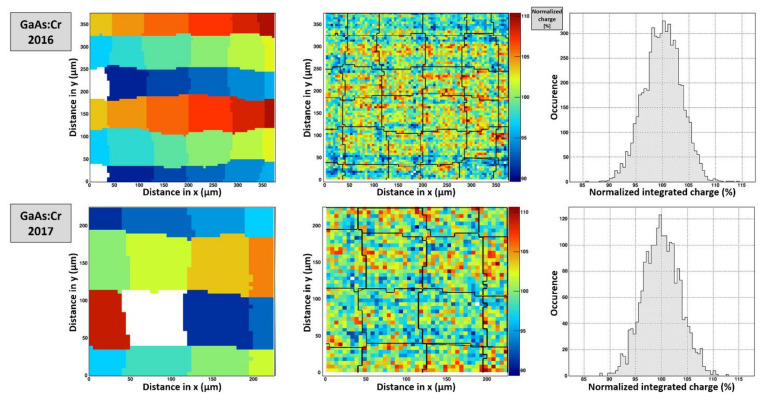
(**left**) The left plots show the merged pixel response map. At each scan point, the pixel with the highest charge is assigned as collecting pixel. (**center**/**right**) Charge collection map and histogram indicating the overall collected charge in all pixels for each scan point. The black lines are the overlaid pixel boundaries extracted from the photon counting representation (with an estimated error margin of ±5 μm).

In order to investigate, how the charge is distributed at the different scan positions, the pixel response maps have been merged. For each point of the raster scan, the collected signal of each pixel was measured and the pixel with the highest signal assigned, indicated by different colors (Figure 19, left).

The response maps show that the effective pixel size in both GaAs:Cr sensors is strongly varied from pixel to pixel. Unfortunately, due to the complexity of the setup and the long duration needed for each measurement and thus the lacking statistics, no quantitative comparison can be done. However, it seems that the homogeneity of the pixel size in sensors from 2017 is better compared to the material from 2016.

Moreover, charge collection maps were created by summing up the charge collected by all pixels in the ROI at each raster scan point. Figure 19 (center) shows the overall collected charge at each scan point together with overlays of the pixel boundaries extracted from the pixel response maps (Figure 19, left). The overall charge appears to be constant throughout the region of interest with a symmetric distribution and a r.m.s. of around 3.5% (Figure 19, right). As the charge is conserved and the penetration depth of the 20 keV photons is relatively shallow, the generated charge carriers seem to move laterally while drifting to the readout electrode.

This variation of the effective pixel size in chromium compensated GaAs was also observed by other groups like J. Becker et al. [13]. The cause of this effect is unknown at this moment; however, it could explain the non-symmetric shape of the charge collection behavior in Figure 14, but this is speculative. More measurements need to be carried out, covering a bigger region of interest and probing how the charge is collected when created at defined sensor depths (e.g., with protons).

## 4. Summary

The characterization results from chromium compensated GaAs sensors from Tomsk State University based on low resistivity LEC-grown wafers have been reported in this paper. The tool for the characterization of the GaAs:Cr sensors is the charge-integrating readout chip JUNGFRAU developed by PSD Detector Group at the Paul Scherrer Institut (PSI).

Table 3 summarizes the characterization results presented in the paper and compared them with results previously obtained with LEC-grown GaAs:Cr sensors obtained from a different vendor #1 and processed in 2016. The dark current in the sensor from 2017 (vendor #2) is 47% lower compared to the sensor from 2016 (vendor #1) with a total current of 13.19 µA, yielding a resistivity of 1.69 × 10^9^ Ω/cm with a dispersion of 17.3% (r.m.s.) over the pixel matrix. The GaAs:Cr (2017) sensors exhibit a noise performance of (101.65 ± 0.04) e^−^ ENC (±9.4% r.m.s.) which is 14% lower than the noise performance of the previously characterized sensor. Improvements can also be reported for the charge transport properties for electrons measured using the Hecht relationship (GaAs:Cr (2017): (μ·τ)_e_ = (4.730 ± 0.003) × 10^−4^ cm^2^/V vs. GaAs:Cr (2016): (μ·τ)_e_ = (1.831 ± 0.002) × 10^−4^ cm^2^/V). In both cases no hole signal could be extracted. The spectral resolution of a monochromatic 60 keV peak obtained with the GaAs:Cr sensor from 2017 is considerably better (FWHM = 2.58 keV or 4.3%) than the value measured with the material from 2016 (FWHM = 4.14 keV or 6.9%), likely due to the improved *µ·τ* product, the lower noise due to a lower dark current and (possibly) the more homogeneous effective pixel size of the material from 2017.

The variation of the effective pixel size of the GaAs:Cr sensors has to be followed up in upcoming measurement campaigns in order to be able to better quantify the effect und find the underlying cause. Furthermore, its consequences on the imaging capability of GaAs:Cr sensors and on position interpolation techniques (where signal heights of neighboring pixel are compared) will be examined.

The origin of the crater effect could be assigned to the short hole lifetime in chromium compensated GaAs which results in a lacking contribution of the hole signal to the overall signal induction. The lifetime of holes for GaAs:Cr (2017) sensors could be determined to be τh = 2.5 ns (compared to τh = 1.4 ns for GaAs:Cr (2016)). Exploiting this effect might be an interesting characterization technique, as it is possible to obtain charge carrier lifetimes that are significantly shorter than the drift time through the sensor in semiconductor sensors with highly differing charge carrier properties.

In general, an improvement of the detector properties was found for the later batches of chromium compensated GaAs sensors, which is mainly reflected in the increase of the resistivity, while still maintaining (and even improving) the charge carrier transport properties. The results presented in this publication indicate that GaAs:Cr is an very interesting sensor material for an application as x-ray detector at synchrotrons and FELs.

However, the dark current is still one of the factors limiting its application as sensor material for charge-integrating detectors when aiming at a good spectral performance. The maximum integration time is defined mainly by the sensor temperature and thus the effort to prevent condensation. In order to achieve full duty cycles at synchrotron sources with integration times of several hundreds of μs, the high dark current poses a severe limitation. This is less problematic at FELs, where the integration times are in the order of few μs.

The short hole lifetime, which leads to the previously described “crater effect”, results in a different detector response depending on the absorption position of the photon in the sensor. The consequences, such as the negative signal in adjacent pixels and the reduced signal in the collecting pixel, can be measured for photons absorbed up to 150–200 μm away from the readout electrode. It should be noted that these effects are also present in single photon counting devices as this is a sensor effect, which leads to unreliable counting behavior due to (effectively) a change of the threshold value. The imminent workaround is limiting the maximum photon energy or by using thicker sensors in order to minimize the number of photons absorbed in this region.

GaAs:Cr sensors might be especially interesting for FELs, as the high dark currents are not a limiting factor due to the short integration times and the maximum photon energies are in a range, where most of the photons will be absorbed close to the backside. Therefore, their use at FELs will be evaluated in future measurement campaigns, using full sized modules consisting of two GaAs:Cr quad size sensors, totaling eight JUNGFRAU1.1 readout chips.

## Figures and Tables

**Table 1:** Charge carrier transport properties of chromium compensated GaAs:Cr (2017) sensors from vendor #2. The parameter space of the electrons was confined by the product of mobility and lifetime that was obtained by the Hecht measurements (μ·τ) _e_ = (4.730 ± 0.003) × 10^−4^ cm^2^/V (±20%).

	Mobility	Lifetime
Electrons	4000 cm^2^/(V·s)	120 ns
Holes	200 cm^2^/(V·s)	2.5 ns

**Table 2:** Estimated drift length, induced signal in the collecting pixel and inducted signal in the eight neighboring pixels of the charge carriers generated in each pixel calculated using the Shockley-Ramo theorem.

Pixel	Distance to Collecting Electrode (z)	Induced Signal in the Collecting Pixel	Induced Signal in the Eight Adjacent Pixels
1	500–490 µm	100.0%	0.0%
2	500–450 µm	100.0%	−0.1%
3	450–365 µm	99.9%	−0.7%
4	365–280 µm	99.7%	−2.0%
5	280–195 µm	99.3%	−4.3%
6	195–110 µm	98.2%	−10.5%
7	110–25 µm	91.9%	−25.1%
8	40–0 µm	77.0%	−34.4%

**Table 3:** Summary of the characterization results from the GaAs:Cr sensors based on LEC-grown wafers from different suppliers. The measurements have been performed under their standard operating parameters of T = +15 °C and a sensor bias voltage of U_Sensor_ = −300 V.

	GaAs:Cr (2017) (Vendor #2)	GaAs:Cr (2016) (Vendor #1)
Dark Current Through Bulk	13.38 µA	25.12 µA
Resistivity	1.69 × 10^9^ Ω/cm (±17.3% r.m.s.)	0.85 × 10^9^ Ω/cm (±15.1% r.m.s.)
Noise (e^−^ ENC, r.m.s) (at t_int_ = 5 μs)	(101.65 ± 0.04) e^−^ ENC (±9.4% r.m.s.)	(115.93 ± 0.03) e^−^ ENC (±5.5% r.m.s.)
FWHM (60 keV)	2.58 keV or 4.3%	4.14 keV or 6.9%
(*µ·τ*) _e_ (by Hecht)	(4.730 ± 0.003) × 10^−4^ cm^2^/V	(1.831 ± 0.002) × 10^−4^ cm^2^/V
CCE at U _HV,Sensor_ = −300 V	98.2%	96.0%
Hole Lifetime τ_h_	2.5 ns	1.4 ns

## Data Availability

Data available on request.

## References

[1] Berger M.J., Hubbell J.H., Seltzer S.M., Chang J., Coursey J.S., Sukumar R., Zucker D.S., Olsen K. (2010). XCOM: Photon Cross Section Database (Version 1.5).

[2] Hamann E. (2013). Characterization of High Resistivity GaAs as Sensor Material for Photon Counting Semiconductor Pixel Detectors. Ph.D. Thesis.

[3] Cola A., Farella I. (2009). The polarization mechanism in CdTe Schottky detectors. Appl. Phys. Lett..

[4] Greiffenberg D. (2010). Charakterisierung von CdTe-Medipix2-Pixeldetektoren. Ph.D. Thesis.

[5] Sellin P. (2003). Recent advances in compound semiconductor radiation detectors. Nucl. Instrum. Methods A.

[6] Harding W.R., Hilsum C., Moncaster M.E., Northrop D.C., Simpson O. (1960). Gallium Arsenide for γ-Ray Spectroscopy. Nature.

[7] Ayzenshtat G., Budnitsky D., Koretskaya O., Novikov V., Okaevich L., Potapov A., Tolbanov O., Tyazhev A., Vorobiev A. (2002). GaAs resistor structures for X-ray imaging detectors. Nucl. Instrum. Methods A.

[8] Tyazhev A., Budnitsky D., Koretskay O., Novikov V., Okaevich L., Potapov A., Tolbanov O., Vorobiev A. (2003). GaAs radiation imaging detectors with an active layer thickness up to 1 mm. Nucl. Instrum. Methods A.

[9] Veale M., Bell S., Duarte D., French M., Schneider A., Seller P., Wilson M., Lozinskaya A., Novikov V., Tolbanov O. (2014). Chromium compensated gallium arsenide detectors for X-ray and γ-ray spectroscopic imaging. Nucl. Instrum. Methods A.

[10] Tlustos L., Shelkov G., Tolbanov O.P. (2011). Characterisation of a GaAs(Cr) Medipix2 hybrid pixel detector. Nucl. Instrum. Methods A.

[11] Veale M.C., Bell S.J., Duarte D.D., French M.J. (2014). Investigating the suitability of GaAs:Cr material for high flux X-ray imaging. J. Instrum..

[12] Hamann E., Koenig T., Zuber M., Cecilia A. (2015). Investigation of GaAs:Cr Timepix assemblies under high flux irradiation. J. Instrum..

[13] Becker J., Tate M.W., Shanks K.S., Philipp H.T. (2018). Characterization of Chromium Compensated GaAs as an x-ray Sensor Material for Charge-Integrating Pixel Array Detectors. J. Instrum..

[14] Veale M.C., Booker P., Cline B., Coughlan J. (2017). MHz rate X-ray imaging with GaAs:Cr sensors using the LPD detectors system. J. Instrum..

[15] Budnitsky D., Tyazhev A., Novikov V., Zarubin A., Tolbanov O., Skakunov M., Hamann E., Fauler A., Fiederle M., Procz S. (2014). Chromium-compensated GaAs detector material and sensors. J. Instrum..

[16] Smolyanskiy P., Bergmann B. (2018). Properties of GaAs:Cr-based Timepix detectors. J. Instrum..

[17] Greiffenberg D., Andrä M., Barten R., Bergamaschi A. (2019). Characterization of GaAs:Cr sensors using the charge-integrating JUNGFRAU readout chip. J. Instrum..

[18] Mozzanica A., Andrä M., Barten R., Bergamaschi A. (2018). The JUNGFRAU Detector for Applications at Synchrotron Light Sources and XFELs. Synchrotron Radiat. News.

[19] Mozzanica A., Bergamaschi A., Brueckner M., Cartier S., Dinapoli R., Greiffenberg D., Tinti G. (2016). Characterization results of the JUNGFRAU full scale readout ASIC. J. Instrum..

[20] Chsherbakov I., Kolesnikova I., Lozinskaya A., Mihaylov T. (2017). Electron mobility-lifetime and resistivity mapping of GaAs:Cr wafers. J. Instrum..

[21] Hamann E., Koenig T., Zuber M., Cecilia A. (2015). Performance of a Medipix3RX Spectroscopic Pixel Detector with a High Resistivity Gallium Arsenide Sensor. IEEE Trans. Med. Imaging.

[22] BM05 Characteristics. http://www.esrf.eu/files/live/sites/www/files/UsersAndScience/Experiments/XNP/BM05/BM05characteristics.pdf.

[23] Hecht Z.K. (1932). Mechanismus des lichtelektrischen Primärstromes in isolierenden Kristallen. Z. Physik A Hadron. Nucl..

[24] Greiffenberg D. (2011). Energy resolution and transport properties of CdTe-Timepix-Assemblies. J. Instrum..

[25] Riegler W., Rinella G.A. (2014). Point charge potential and weighting field of a pixel or pad in a plane condenser. Nucl. Instrum. Methods A.

[26] Zarubin A.N., Mokeev D.Y., Okaevich L.S., Tyazhev A.V., Bimatov M.V., Lelekov M.A., Ponomarev I.V. (2006). Non-equilibrium Charge Carriers Life Times in Semi-Insulating GaAs Compensated with Chromium. Int. Workshops Tutor. Electron Devices Mater..

